# Remifentanil inhibits the traumatic stress response in emergent trauma surgery

**DOI:** 10.1002/jcla.22971

**Published:** 2019-08-02

**Authors:** Ru Ouyang, Haijing Ren, Wei Liu, Xi Yuan, Enjun Lei

**Affiliations:** ^1^ Department of Anesthesiology The First Affiliated Hospital of Nanchang University Nanchang China; ^2^ Department of Medicine, Graduate School Nanchang University Nanchang China

**Keywords:** emergent surgery protection, remifentanil, stress response, sufentanil, trauma

## Abstract

**Objective:**

The aim of this study was to explore whether remifentanil could inhibit the stress response in emergent trauma surgery more effectively than sufentanil.

**Patients and methods:**

Sixty trauma patients for emergent surgery were randomly divided into remifentanil group (R group, n = 30) or sufentanil group (S group, n = 30). The patients in the R group were continuously intravenously infused with remifentanil, while those in the S group were administrated with sufentanil. The plasma contents of cortisol (COR), epinephrine (E), norepinephrine (NE), and blood glucose were measured before anesthesia induction (T1), 5 minutes after intratracheal intubation (T2) and 5 minutes (T3), 30 minutes (T4), and 1 hour (T5) after surgery, respectively. The blood pressure (BP) and the heart rate (HR) at these time points were recorded as well.

**Results:**

The results showed that the patients in the R group had more stable hemodynamics during the surgery and had a significantly lower HR at T2‐T5 than those in the S group. The plasma levels of norepinephrine at time points T3‐T5 and levels of cortisol at T4‐T5 in the R group were significantly lower than those in the S group (*P* < 0.05).

**Conclusions:**

The results in the present study indicated that remifentanil could inhibit the stress response in emergent trauma surgery patients more effectively than sufentanil.

## 1 INTRODUCTION

In recent years, with the rapid development of transportation, industry, construction, etc, the occurrence of various major accidents has increased dramatically. Traffic accidents have become the main cause of trauma, and trauma has become one of the main causes of global death.^1^ More than 5.5 million people die from traumatic accidents each year all over the world, accounting for 11.87% of the total mortality rate.^2^ Surgery is one of the most important therapeutic strategies for patients with severe trauma. However, both anesthesia and surgical operation can cause the stress responses, and a severe stress response can further lead to immune dysfunctions; these processes would eventually affect the long‐term efficacy of the treatment and prognosis of the patient.^3^, ^4^ Therefore, effective inhibition of the intraoperative stress response is critical for patients with trauma. Although many studies have revealed that remifentanil can inhibit sympathetic nerve excitement,^5^, ^6^, ^7^ whether remifentanil can inhibit the stress response more effectively than sufentanil in trauma patients undergoing emergent surgery is still unclarified. In our study, we respectively compared the serum levels of adrenaline, norepinephrine, cortisol, and glucose in traumatic patients undergoing emergent surgery under general anesthesia with continuous infusion with remifentanil or sufentanil. Furthermore, the changes in HR and BP of patients in these two groups were also compared. Thus, the goal of this study was to clarify whether remifentanil could inhibit the stress response in trauma patients undergoing emergent surgery more effectively than sufentanil.

## 2 PATIENTS AND METHODS

### 2.1 Patients

This study was approved by the Medical Ethics Committee of the First Affiliated Hospital of Nanchang University, and informed consent was signed by the patients. Fifty trauma patients for emergent (28 males and 22 females, 18‐65 years old, mainly for spinal, limb, and maxillofacial injuries) surgery in the First Affiliated Hospital of Nanchang University from May 2017 to May 2018 were included. All patients had no traumatic shock, serious cardio‐cerebral disease, adrenal disease, endocrine or metabolic diseases, or hormonal and vasoactive drugs use history and had normal liver, kidney, and coagulation function. All patients were randomly divided into remifentanil group (R group) or sufentanil group (S group) with 30 cases in each group.

### 2.2 Methods

After venous access was obtained, the radial artery and right internal jugular vein catheter were placed under local anesthesia. Invasive systolic blood pressure (SBP), diastolic blood pressure (DBP), mean arterial pressure (MAP), heart rate (HR), oxygen saturation (SPO2), electrocardiogram (ECG), and bispectral index (Bis) were monitored with a multifunction monitor (DATEX‐OHMEDA, Beijing Haiyongrui Trading Co., Ltd.). Dexmedetomidine (0.8‐1 µg/kg, batch number: 10 020 334, Jiangsu Hengrui Pharmaceutical Co., Ltd.) was intravenously infused into every patient in 10 minutes. Anesthesia was induced with propofol (1.5‐2.0 mg/kg), atracurium (0.6‐0.8 mg/kg), and sufentanil (0.4‐0.6 µg/kg). Then, the patient's trachea was intubated with a tracheal tube, and mechanical ventilation was performed with maintenance of the pressure of end‐tidal carbon dioxide (PETCO2) at 35‐45 mm Hg. The Bis value was maintained at 45‐55 with propofol, 8 ~ 10 µg/kg/h remifentanil and cisatracurium for the patients in the R group, whereas those in the S group were maintained with propofol, 0.02‐0.04 µg/kg/min sufentanil and cisatracurium. The infusion of remifentanil was stopped at the end of surgery, while the infusion of sufentanil was stopped at 20 minutes before surgery. At the end of the operation, 50 mg flurbiprofen ester was given to all the patients in both groups for analgesia. After anesthesia recovery, patient‐controlled intravenous analgesia was performed for all patients in the two groups.

### 2.3 Observation indexes

Three milliliters of the internal jugular vein blood were collected from all patients before anesthesia induction (T1), 5 minutes after intratracheal intubation (T2) and 5 minutes (T3), 30 minutes (T4), and 1 hour (T5) after the start of surgery, respectively. The blood pressure (BP) and heart rate (HR) at these time points were also recorded. The blood was centrifuged at 2500 rpm/min for 20 minutes, and the plasma was collected. The levels of epinephrine (E), norepinephrine (NE), and plasma cortisol (COR) in plasma were measured by radioimmunoassay. Blood glucose (GLU) was measured by the oxidase method.

### 2.4 Statistical analysis

Statistical Product and Service Solutions (SPSS) 20.0 statistical analysis software (IBM) was used to analyze the data. The data were expressed as the mean ± standard deviation. All the data were analyzed by repeated measurement two‐way analysis of variance with prenatal treatment as the between‐subjects independent factor and time point as the repeated factor with the least significant difference post hoc test. A difference with a *P* < 0.05 was considered statistically significant.

## 3 RESULTS

### 3.1 Comparison of the basic clinical data between the two groups

There were no statistically significant differences in age, gender, weight, type of trauma, or operation time between the two groups (*P* > 0.05) (Table 1).

**Table 1:** Comparison of the basic clinical data between two groups

Group	BMI (kg/m^2^)	Trauma	Type			Operation time (min)
		Spinal trauma	Lower extremity trauma	Upper limb trauma	Maxillofacial trauma	
R Group	22.5 ± 3.2	9	12	6	3	120 ± 18.7
S Group	22.3 ± 3.6	11	10	7	2	124 ± 15.2

Abbreviation: BMI, body mass index.

### 3.2 Comparison of hemodynamics between the two groups

Compared with the MAP and HR at T1, the MAP and HR at time points T2‐T5 were significantly lower in both groups (*P* < 0.05). The MAP and HR at T1 have no significant differences between the two groups (*P* > 0.05), whereas both the MAP and HR at time points T2‐T5 in the R group were significantly lower than those in the S group (*P* < 0.05) (Table 2).

**Table 2:** Comparison of hemodynamics between two groups

Index	Group	T1	T2	T3	T4	T5
MAP (mm Hg)	R Group	104.07 ± 6.73	79.27 ± 4.59*, **	79.43 ± 4.14*, **	78.07 ± 4.20*, **	78.93 ± 3.62*, **
	S Group	102.90 ± 4.80	86.77 ± 3.96**	86.30 ± 3.44**	56.90 ± 3.36**	87.73 ± 3.61**
HR (Beat/min)	R Group	97.47 ± 11.46	69.10 ± 5.79*, **	59.37 ± 5.99*, **, ***	57.07 ± 5.11*, **, ***	58.17 ± 5.76*, **, ***
	S Group	97.47 ± 13.27	74.11 ± 4.45**	73.83 ± 4.11**	74.63 ± 3.44**	74.37 ± 3.59**

* *P* < 0.05, compared with the value in group S at the same time point.

** *P* < 0.05, intragroup comparisons with the baseline value at T1.

*** *P* < 0.05, intragroup comparisons with the baseline value at T2.

The MAP at T3‐T5 in the two groups was not significantly changed than the MAP at T2 (*P* > 0.05). However, the HR of patients in the R group at T3‐T5 was significantly lower than that in the S group (*P* < 0.05) (Table 3).

**Table 3:** Changes in plasma markers at relevant time points

Index	Group	T1	T2	T3	T4	T5
E (pg/mL)	R Group	160.46 ± 44.32	142.53 ± 43.94	132.93 ± 39.37**	123.65 ± 38.18*, **	117.36 ± 37.84*, **
	S Group	161.37 ± 45.01	150.27 ± 43.85	145.09 ± 41.75	145.34 ± 41.51	146.00 ± 42.44
NE (ng/mL)	R Group	2.16 ± 0.80	1.98 ± 0.75	1.77 ± 0.72	1.67 ± 0.62	1.57 ± 0.59
	S Group	2.17 ± 0.81	2.05 ± 0.76	1.83 ± 0.73	1.70 ± 0.69	1.64 ± 0.71
COR (ng/mL)	R Group	249.98 ± 68.57	227.16 ± 79.68	221.18 ± 76.64	190.30 ± 75.54**	172.17 ± 75.26*, **
	S Group	251.61 ± 84.31	242.06 ± 85.35	226.46 ± 82.92	217.06 ± 71.64	214.72 ± 82.15
GLU (mmol/mL)	R Group	8.97 ± 2.86	6.97 ± 1.89**	6.94 ± 1.88**	6.83 ± 1.87**	7.00 ± 1.65**
	S Group	8.99 ± 2.06	7.29 ± 1.59**	7.71 ± 1.50**	7.76 ± 1.52**	7.71 ± 1.46**

* *P* < 0.05, compared with the value in group S at the same time point

** *P* < 0.05, intragroup comparison with the baseline value at T1.

### 3.3 Comparison of plasma markers between the two groups

Compared with the epinephrine level at T1, the levels of epinephrine at T3‐T5 in the R group were significantly lower (*P* < 0.05), while those in the S group were not significantly changed from the level at T1 (*P* > 0.05). The levels of epinephrine at T4‐T5 in the R group were significantly lower than those in the S group (*P* < 0.05). In the R group, the cortisol levels at T4‐T5 were significantly lower than that at T1 (*P* < 0.05), whereas there was no significant difference observed in the S group (*P* > 0.05). The level of cortisol at T5 in the R group was significantly lower than that in the S group (*P* < 0.05). The blood glucose levels at T2‐T5 in the two groups were significantly lower than those at T1 (*P* < 0.05). The levels of norepinephrine at T2‐T5 in the two groups have no significant differences compared with the level of norepinephrine at T1 (*P* > 0.05). There was no significant difference in NE levels among all time points in the two groups or between the two groups (*P* > 0.05). The levels of GLU at T2‐T5 in the two groups were lower than those at T1 (*P* < 0.05). There was no significant difference in the levels of NE or GLU at any of the observed time points between the two groups (*P* > 0.05) (Table 3).

## 4 DISCUSSION

The results of the present study showed that remifentanil induced more stable hemodynamics, lower plasma levels of norepinephrine, and cortisol than sufentanil in emergent trauma surgery patients. These results indicated that remifentanil could inhibit the stress response in emergent trauma surgery patients more effectively than sufentanil did.

As is known, trauma can activate a series of neuroendocrine reactions with sympathetic excitation and hypothalamic‐pituitary‐adrenal axis secretion and then cause various functional and metabolic changes.^8^ The stress response mainly manifests as neuroendocrine dysfunction, such as the enhancement of the plasma AD and NE levels, the excessive secretion of hormones or the lacking of hormone synthesis; thus the stress response can be observed as the increment of blood pressure, blood sugar, heart rate etc.^9^, ^10^ Surgery is an important treatment for trauma patients. Appropriate inhibition of the intraoperative stress response is critical for patients undergoing surgeries.^11^ Remifentanil has been confirmed to have excellent antioxidative properties, as well as to inhibit the production of inflammatory factors and alleviate the damage of tissue and organ reperfusion injury.^12^, ^13^, ^14^

The present results showed that remifentanil induced more stable hemodynamics than sufentanil.^15^, ^16^ This difference in hemodynamics may be associated with the more effective dose‐dependent inhibition of sympathetic activity by remifentanil than by other opioids.^17^, ^18^

Cortisol, adrenaline, and norepinephrine are the main hormones released during the processes of stress response. The results of this study showed that the plasma levels of E, NE, and COR in both groups began to decrease after anesthesia, and those in the R group became lower than those in the S group (*P* < 0.05). These results are consistent with previous reports.^19^, ^20^ The levels of NE or glucose have no significant differences between the two groups (*P* > 0.05), suggesting that the traumatic emergency patients had a strong stress response and the NE release may be related to the central norepinephrine activation system. Furthermore, these results suggested that remifentanil does not completely inhibit the central norepinephrine system.

Meta‐analysis results have indicated that the permissive hypotension could reduce bleeding and blood product use and reduce mortality when organ perfusion is ensured.^18^ Mediha TÜRKTAN^21^ and other studies have shown that intravenous target‐controlled infusion of propofol, remifentanil, and dexmedetomidine reduced intraoperative bleeding and improved surgical outcomes. Controlled hypotension with remifentanil has also been shown to be able to stabilize hemodynamics and reduce blood loss^22^, ^23^ with a stable cardiac index and effective tissue perfusion.^24^

In summary, remifentanil inhibits the stress response during traumatic emergency surgery by reducing the release of catecholamines and cortisol more effectively than sufentanil.

## 5 CONCLUSIONS

The present study indicated that remifentanil could inhibit the stress response in emergent trauma surgery patients more effectively than sufentanil.
